# Clinical Case Report: Neonatal Mesenteric Traction Syndrome During Surgery for Congenital Duodenal Stenosis

**DOI:** 10.7759/cureus.63994

**Published:** 2024-07-06

**Authors:** Sahoko Kodama, Maiko Tomiki, Koji Sato, Shigeharu Ueki, Yukitoshi Niiyama

**Affiliations:** 1 Department of Anesthesiology and Intensive Care Medicine, Akita University Graduate School of Medicine, Akita, JPN; 2 Department of General Internal Medicine and Clinical Laboratory Medicine, Akita University Graduate School of Medicine, Akita, JPN

**Keywords:** surgery, neonate, anaphylactoid reaction, general anesthesia, mesenteric traction syndrome

## Abstract

Mesenteric traction syndrome (MTS) is a common complication of major abdominal surgery, characterized by flushing, hypotension, and tachycardia. However, its occurrence in neonates has not yet been documented. This report details a neonatal case of MTS that emerged during surgery for congenital duodenal stenosis. The patient was a two-day-old girl, born at 39 weeks and three days of gestation via vaginal delivery, weighing 2708 g. She underwent general anesthesia for the surgery, and continuous IV administration of remifentanil at 0.2 μg/kg/min was commenced minutes before the surgery began. Initially, her arterial pressure was 60-70/40-50 mmHg. However, shortly after bowel manipulation began, her blood pressure rapidly declined to 31/25 mmHg. Concurrently, her heart rate increased from 120 to 170 beats per minute, and she displayed facial and upper body flushing. Arterial blood gas analysis indicated a PaO2 drop from 124 to 61 mmHg at an FiO2 of 0.3. Treatment consisted of infusion loading and two bolus doses of 2.5 μg of phenylephrine (diluted to 2.5 μg/mL), which normalized her blood pressure within approximately 10 minutes. The facial flushing gradually subsided and disappeared after 40 minutes. Subsequent circulatory stability allowed for the successful completion of the surgery. There was no alteration in airway pressure, and hemodynamic stability was only compromised following the commencement of bowel manipulation. Given the serious risks associated with prolonged hemodynamic instability in neonates, the potential for MTS should be considered during anesthetic management in such cases.

## Introduction

Mesenteric traction syndrome (MTS), a common complication following major abdominal surgery, is characterized by symptoms such as flushing, hypotension, and tachycardia. Currently, there are no clear diagnostic criteria for MTS; the diagnosis is based on symptoms and the exclusion of other conditions. In the perioperative period, it is important to differentiate anaphylactic reactions from conditions related to muscle relaxants or antibiotics. MTS is associated with intra-abdominal procedures and typically occurs about 20 minutes after the start of surgery. Hemodynamics usually stabilize within 60 minutes with the symptomatic administration of IV fluids and vasoconstrictors, but circulatory compromise may occur in critically ill patients. MTS occurs in more than 70% of adults who undergo laparotomy [1]; however, few reported cases involve pediatric patients. In this report, we detail a neonatal case of MTS during laparotomy.

## Case presentation

Clinical background

A two-day-old girl, born vaginally at 39 weeks and 3 days of gestation and weighing 2708 g, was brought to the operating room for duodenal stenosis surgery. Her Apgar scores were 8 at 1 minute and 9 at 5 minutes. She exhibited no unusual facial features or physical abnormalities, including those suggestive of chromosomal abnormalities. Prenatal ultrasound surveillance had revealed a dilated upper GI tract, leading to a postnatal diagnosis of congenital duodenal stenosis via GI radiography. Echocardiography suggested a ventricular septal defect; however, the patient showed no circulatory issues, and spontaneous closure was anticipated. The results of preoperative laboratory tests, including liver and renal function, serum electrolyte levels, glucose levels, peripheral blood counts, and blood gas analysis, were all within normal ranges. The patient was prepared for surgery under general anesthesia.

Operation and anesthesia

**Figure 1:**
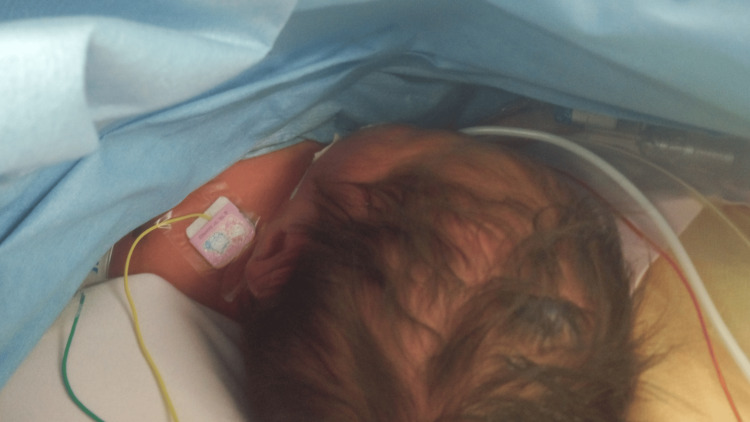
Skin flushing on the patient’s upper body.

Upon admission to the operating room, the patient’s vital signs were: body temperature of 37.3°C, heart rate of 130-140 beats per minute, and blood pressure of 86/31 mmHg. Following admission, anesthesia was induced under standard monitoring conditions. The rapid induction protocol involved administering 15 mg of thiopental and 3 μg of fentanyl, followed by 2.5 mg of rocuronium for muscle relaxation. An inner diameter (ID) 3.0 mm tracheal tube, equipped with a cuff, was then orally inserted. After induction, IV access was established in the right hand, and an arterial line was secured to the left radial artery. Anesthetic maintenance involved administering oxygen at 1-2 L/min, air at 2-4 L/min, and sevoflurane at 1.0%-1.5%. A continuous IV infusion of remifentanil was started at 0.2 μg/kg/min, initiated shortly before the surgical procedure. The immediate preoperative assessment revealed an arterial pressure range of 60-70/40-50 mmHg. However, approximately 10 minutes after bowel manipulation began, the blood pressure started to decline rapidly, reaching a nadir of 31/25 mmHg. Concurrently, the heart rate surged from 120 to 170 beats per minute. Observation revealed marked facial and upper body skin flushing (Figure 1).

**Figure 2:**
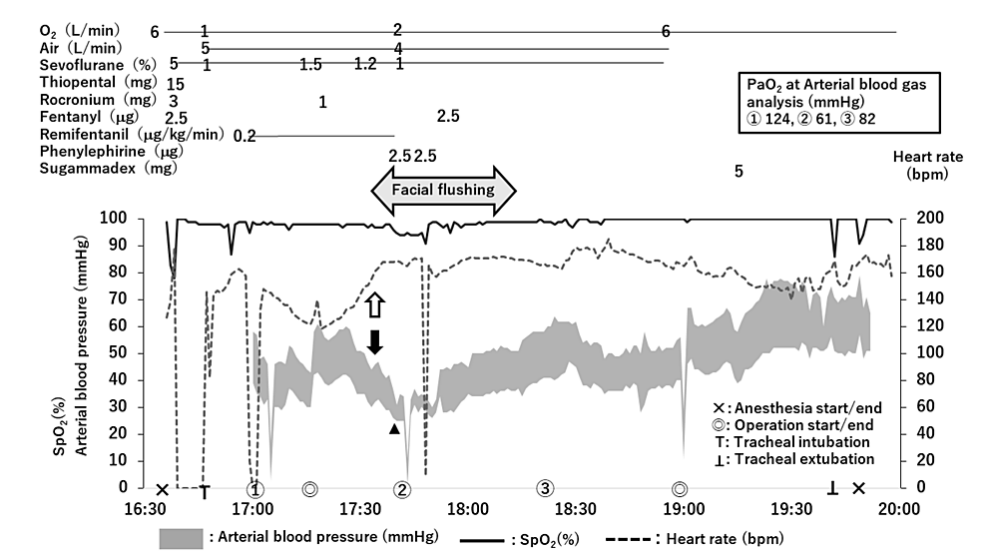
Anesthesia record. General anesthesia was initiated using thiopental, fentanyl, and rocuronium. Following tracheal intubation, anesthesia was maintained with sevoflurane and remifentanil. Shortly after surgery began, the patient experienced a rapid decrease in blood pressure (➡), reaching a low of 31/25 (28) mmHg (▲), accompanied by an increase in heart rate from 120 to 170 beats per minute (⇨). Concurrently, facial flushing occurred, lasting approximately 40 minutes before resolving spontaneously. The patient’s hemodynamics stabilized within approximately 20 minutes following vasopressor administration and fluid infusion, enabling the successful completion of the surgery. Shaded area represents arterial blood pressure; dotted line represents heart rate; solid line represents percutaneous oxygen saturation; ✕: anesthesia start/end, ◎: operation start/end, Τ: tracheal intubation, ⊥: tracheal extubation.

The blood pressure normalized within 10 minutes following the administration of extracellular fluid and albumin preparation, along with two bolus doses of 2.5 μg of phenylephrine (diluted to 2.5 μg/mL). The patient's PaO2 dropped from 124 to 61 mmHg at an FiO2 of 0.3, improving concurrently with the blood pressure. The flushing subsided and disappeared approximately 40 minutes later. Subsequently, the patient’s circulatory condition stabilized, allowing for the successful completion of the duodeno-jejunostomy surgery. The anesthesia record is shown in Figure 2.

## Discussion

MTS represents a syndrome characterized by pronounced circulatory disturbances (hypotension, tachycardia), along with facial flushing and palmar erythema, instigated by mesenteric traction during laparotomy. This syndrome is believed to arise from endothelial cell shear stress in the mesenteric vessels caused by traction on the small intestine and mesentery, activating cyclooxygenase, which leads to prostacyclin production and systemic vasodilation [2,3].

The incidence of MTS in children has not been clearly documented. As previously stated, MTS has a high incidence in adults undergoing open abdominal surgery, and its mechanism of occurrence may be similar in children. However, because many patients improve within 30-60 minutes with symptomatic treatment, it is possible that MTS has occurred in children but has not been reported.

In neonates, especially in the immediate postnatal period, hepatic and renal functions are particularly immature, and the effects of many medications may be prolonged. However, the levels of the nonspecific esterases that metabolize remifentanil are almost as high in neonates as in adults, and the use of remifentanil is increasing in neonates because postoperative respiratory depression is less likely to occur [4]. Standardized guidelines for the use of remifentanil in pediatric settings are lacking, leading to variations in practice based on individual anesthesiologists’ discretion and facility-specific nuances. In this case, it was necessary to administer strong analgesia during the surgical procedure. Remifentanil was selected as the optimal agent for this purpose because it has the shortest duration of action, with minimal concern for postoperative respiratory depression and delayed arousal due to residual opioids.

Nomura Y et al. [5] reported an increased incidence of MTS associated with the use of remifentanil during abdominal surgery. The authors stated that remifentanil’s sympathomimetic depressive properties and calcium channel-blocking effects on the mesenteric vasculature may induce vasodilation, thereby contributing to the development of MTS. However, the pathophysiological mechanisms remain under investigation. Although no clear diagnostic criteria for MTS currently exist, one diagnostic marker is 6-keto-prostaglandin F1α (6-keto-PGF1α), a stable metabolite of prostacyclin. Previous studies have demonstrated a significant correlation between 6-keto-PGF1α concentrations and several cardiovascular parameters, including systemic vascular resistance, heart rate, cardiac output, and facial flushing, in the context of mesenteric traction [6,7]. Nevertheless, the relationship between remifentanil and 6-keto-PGF1α concentrations remains unclear, and further studies are anticipated to elucidate this relationship.

The potencies of fentanyl and remifentanil are nearly equivalent, and they are purported to have comparable analgesic effects at the same effect site concentration. The dose rate of remifentanil in our patient was not particularly high, but the effect site concentration of remifentanil increased more rapidly than that of fentanyl, and the onset of action was faster. Consequently, the effect site concentration may have been higher than anticipated at the start of the surgery, which may have resulted in a stronger vasodilatory effect. It is therefore possible that remifentanil had a direct effect or increased vasodilation, which may have precipitated the development of MTS in this case.

MTS typically improves within 30 to 60 minutes following the administration of intravenous fluids and vasoconstrictors, as observed in this case. We employed the α1 receptor stimulant phenylephrine; if this had been ineffective, however, we may have considered noradrenaline, a more potent vasoconstrictor. Furthermore, the infusion of additional extracellular fluid, hydroxyethyl starch, and albumin preparations may be considered to augment the circulating blood volume. For patients at risk of significant circulatory instability in the event of severe MTS, as in this case, it may be beneficial to provide adequate preoperative fluids. However, clinicians must exercise caution and avoid excessive perioperative infusions because they can lead to complications such as bowel edema, which can in turn result in suture failure and increased abdominal pressure after abdominal closure.

Takahashi H et al. [8] suggested the intravenous administration of flurbiprofen, a cyclooxygenase inhibitor, as a potential method to reduce the incidence of MTS in adults. As indicated in the accompanying text, flurbiprofen is not contraindicated in pediatric patients. In this case, it was not administered due to the lack of experience with its use in neonates. If flurbiprofen is administered, it is of paramount importance to monitor the patient for the development of allergic reactions and respiratory symptoms such as wheezing and dyspnea. Furthermore, circulatory instability may be exacerbated by histamine release from mast cells in the mesentery, caused by stretching of the small intestine. Previous research has demonstrated the efficacy of antihistamines in reducing the incidence of arrhythmias in adults and the need for vasoconstrictors and intravenous fluids [9]. Nevertheless, antihistamines have not yet been implemented in practice for these purposes.

MTS, often presenting with early surgical phase facial flushing, hypotension, and tachycardia, should be considered alongside other entities, including anaphylactic reactions. However, the low frequency (0.005%) of anaphylactic reactions under general anesthesia with nondepolarizing muscle relaxants among Japanese patients [10], along with the absence of alterations in airway pressure and the timing of intraperitoneal manipulation, makes anaphylaxis unlikely in our case. Nevertheless, the possibility of anaphylaxis cannot be completely ruled out. Some pediatric patients require other surgical procedures later in life. Therefore, plasma histamine and tryptase levels should be measured immediately after the onset of symptoms. In addition, the cause of the symptoms should be thoroughly investigated with postoperative skin tests and basophil activation tests, if necessary, to differentiate the symptoms from manifestations of allergy. Other potential pathologies must also be considered. In our patient, the sudden drop in blood pressure may have been attributable to a reduced circulating blood volume or the effects of general anesthesia. However, these factors do not fully explain the pronounced facial flushing. Although the blood levels of prostacyclin and histamine were not measured in this case, therefore remaining unconfirmed, MTS remains the most likely diagnosis considering the overall clinical context and in light of the aforementioned considerations.

Furthermore, a reduction in PaO2 was observed in conjunction with a decline in the intraoperative blood pressure. The abdominal wound was relatively minor and no discernible changes in airway pressure were observed, therefore, it is unlikely to have exerted a significant compression force on the lungs. We hypothesize that the observed decrease in PaO2 was primarily attributable to a reduction in cardiac output resulting from a decline in venous return due to vasodilation.

Currently, there is a lack of evidence supporting the efficacy of MTS prophylaxis in reducing postoperative complications or improving outcomes. Nevertheless, persistent unstable circulation remains a significant risk factor for adverse outcomes in neonates and patients with underlying disease. While milder cases of MTS often follow a benign course, severe manifestations have been linked to increased risks of stroke, myocardial infarction, and adverse postoperative outcomes, possibly due to systemic inflammatory response and vascular endothelial damage induced by surgical stress [11]. These adverse effects are particularly associated with severe forms of MTS. Future research should focus on assessing MTS severity, developing effective treatment strategies, and preventing severe cases, which can also be applied to neonates.

Currently, there are no universally accepted diagnostic criteria for MTS. The diagnosis and grading of MTS are typically based on subjective visual assessments of the degree and extent of facial flushing. The common approach categorizes MTS into three levels: no MTS (absence of flushing), moderate MTS (mild facial flushing), and severe MTS (pronounced facial flushing extending to the shoulder and chest) [1]. However, subjective evaluation lacks consistency between observers and may be further complicated by differences in skin color and conditions such as anemia. Ring LL et al. [7] proposed an objective method using laser speckle contrast imaging to quantitatively measure facial flushing, which could standardize assessments across studies and support multicenter collaborations.

The efficacy of flurbiprofen in treating MTS has been reported [8], but optimal dosing and its effectiveness in the postoperative period require further investigation. Phenylephrine is commonly used as a vasoconstrictor in clinical practice for MTS treatment, but comparative studies with other vasoconstrictors (such as noradrenaline and vasopressin) and investigations into the optimal choice of vasoconstrictor are warranted. Utilizing the Flo Track sensor for monitoring could aid in diagnosing and treating MTS [12]. Monitoring stroke volume variation and systemic vascular resistance index may guide therapeutic decisions regarding vasoconstrictor or infusion therapy selection. Neonates undergo a significant transition from fetal to pulmonary circulation. Preterm and sick neonates may encounter challenges in adapting to these changes and may have a narrow tolerance for them. Therefore, it is crucial to identify the cause of circulatory failure during neonatal surgery and intervene promptly and effectively.

Severe MTS is associated with profound hypotension, which stimulates sympathetic activity, adrenal medulla catecholamine secretion, and elevated endogenous catecholamine levels. Olsen AA et al. [11] demonstrated elevated preoperative epinephrine levels in patients who developed severe MTS, suggesting potential prophylactic interventions targeting preoperative epinephrine levels as a preventive measure. As the patients in this report were adults, it is imperative to ascertain the relevance and applicability of the findings to neonates and children.

The pathophysiology that contributes to the increased rate of postoperative complications seen in patients with severe MTS is complex and remains largely unexplored. Further research is anticipated to focus on establishing objective diagnostic criteria and elucidating the mechanisms of hemodynamic alterations, sympathetic activation, and endothelial cell damage.

## Conclusions

We have herein reported a neonatal case of MTS that developed during the surgical correction of duodenal atresia. Neonates are particularly susceptible to the severe consequences of persistent hemodynamic instability, and clinicians should be aware of the possibility of MTS developing intraoperatively. Furthermore, MTS warrants renewed attention due to its association with systemic inflammation and an unfavorable prognosis.
